# Disruption of Mitochondrion-To-Nucleus Interaction in Deceased Cloned Piglets

**DOI:** 10.1371/journal.pone.0129378

**Published:** 2015-06-11

**Authors:** Joonghoon Park, Liangxue Lai, Melissa S. Samuel, David Wax, Randall S. Prather, Xiuchun Tian

**Affiliations:** 1 Department of Animal Science and Center for Regenerative Biology, University of Connecticut, Storrs, Connecticut, United States of America, 06269; 2 Division of Animal Science, University of Missouri-Columbia, Columbia, Missouri, United States of America, 65211; Utah State University, UNITED STATES

## Abstract

Most animals produced by somatic cell nuclear transfer (SCNT) are heteroplasmic for mitochondrial DNA (mtDNA). Oxidative phosphorylation (OXPHOS) in clones therefore requires the coordinated expression of genes encoded by the nuclear DNA and the two sources of mitochondria. Such interaction is rarely studied because most clones are generated using slaughterhouse oocytes of unrecorded origin. Here we traced the maternal lineages of seven diseased and five one-month-old live cloned piglets by sequencing their mtDNA. Additionally by using a 13K oligonucleotide microarray, we compared the expression profiles of nuclear and mtDNA-encoded genes that are involved in mitochondrial functions and regulation between the cloned groups and their age-matched controls (n=5 per group). We found that the oocytes used to generate the cloned piglets were of either the Large White or Duroc background, and oocyte genetic background was not related to the clones’ survival. Expression profiles of mtDNA-encoded genes in clones and controls showed intermixed clustering patterns without treatment or maternal lineage-dependency. In contrast, clones and controls clustered separately for their global and nuclear DNA-encoded mitochondrial genes in the lungs for both the deceased and live groups. Functional annotation of differentially expressed genes encoded by both nuclear and mtDNA revealed abnormal gene expression in the mitochondrial OXPHOS pathway in deceased clones. Among the nine differentially expressed genes of the OXPHOS pathway, seven were down-regulated in deceased clones compared to controls, suggesting deficiencies in mitochondrial functions. Together, these data demonstrate that the coordination of expression of mitochondrial genes encoded by nuclear and mtDNA is disrupted in the lung of diseased clones.

## Introduction

Since the first report of cloning success in mammals by SCNT [1], mammalian oocytes have been recognized as possessing all the necessary components for complete reprogramming [2]. The number of healthy births, however, is still low and most cloned animals suffer from a variety of abnormal phenotypes [1, 3, 4]. Nevertheless, SCNT is still more efficient in reprogramming capacity than the induced pluripotent stem cell technology, which reprograms in the range of 0.1%.

In the last decade, efforts have been made to compare cloned animals to those of conventional breeding in order to further improve cloning efficiency [5–7]. A major area of difference lies in mitochondria which are important in ATP generation and in programmed cell death [8]. Conventionally bred animals are homoplasmic for the maternal mitochondria, while most SCNT animals are heteroplasmic, containing mitochondria from both the recipient oocytes and the somatic donor cells [9–12].

The mitochondrial genome is composed of 37 genes encoding 13 subunits of the respiratory enzyme complexes in the OXPHOS pathway as well as 22 tRNAs and two rRNAs. The majority of the elements in the OXPHOS pathway as well as other proteins involved in mitochondrial regulation and biogenesis (approximately 1,500 proteins) is encoded by nuclear DNA [13, 14]. Therefore, the coordinated expression of the nuclear and mtDNA and the interaction of the protein products are critical in maintaining normal mitochondrial and host cell functions. Mitochondrial dysfunction also feeds back to the host cell by altering expression of numerous nuclear DNA-encoded genes to readjust its metabolic profile. For example, in yeast, retrograde response from mitochondria to the nucleus influences many cellular activities under both normal and pathophysiological conditions [15]. Likewise, mitochondrial OXPHOS is defective in cytoplasmic hybrids containing rat mtDNA in mouse cells due to impairment of coordinated assembly of nuclear- and mtDNA-encoded OXPHOS subunits [16]. In addition, mutations in mtDNA can induce genomic instability as well as tumorigenesis [17].

Oocytes used in cloning farm animals are usually obtained from slaughterhouses [18], and thus animals cloned from the same donor cell line may not be entirely identical due to the different mtDNA. Heteroplasmy may interfere with the development of the cloned embryos and thus cloning outcome due to improper interaction of the donor nuclear DNA and recipient oocyte’s mitochondria in both intra- and, more likely, inter-species nuclear transfer. Several indirect lines of evidence support such a possibility. For example, reconstructed cytoplasmic hybrid embryos from Bos Taurus (Brown Swiss) granulosa cells and oocytes that contained B. taurus A (Simmental), B. taurus B (Simmental), or Bos indicus (Dwarf Zebu) cytoplasm have different developmental potentials in bovine [19]. Additionally, inter-subspecies SCNT using cytoplasts from oocytes of crossbred goats (Saanen male symbol x Boer female symbol descendant) improves embryo-fetal development than cytoplasts from Saanen oocytes [20]. Furthermore, higher efficiencies were observed in homoplasmic cloning in both cattle and sheep [21, 22]. However, whether mtDNA-encoded genes are coordinately expressed with those encoded by the nucleus has not been studied in clones.

In this study, we used DNA sequencing to identify the maternal lineages of oocytes in cloned pigs and subsequently analyzed the expression profiles of global, nuclear DNA- and mtDNA-encoded genes involved in OXPHOS in deceased and live cloned pigs. We found that expression profiles of mtDNA-encoded genes of clones and controls clustered independently from treatment or maternal lineage. Clones and controls clustered separately for their global and nuclear DNA-encoded mitochondrial genes in the lungs for both deceased and live groups. Functional annotation analyses of differentially expressed genes encoded by the nuclear DNA and mtDNA genes revealed defects in the mitochondrial OXPHOS pathway in deceased clones.

## Materials and Methods

### RNA and DNA isolation

All animal experimentation was conducted at the University of Missouri. All procedures were approved by the University of Missouri Institutional Animal Care and Use Committee. Veterinary staff was consulted on a daily basis regarding the health and care of the cloned piglets, humane sacrifice, and tissue collection.

Frozen lung tissues of cloned piglets by SCNT and of age-matched controls by conventional breeding were provided by Dr. Randall Prather of the University of Missouri-Columbia [4, 23]. Seven clones that died shortly after birth were designated as the “deceased” group, and five one-month old clones as the “live” group. Each cloned group was age-matched with five controls from conventional reproduction. Total RNA was isolated using TRIzol (Invitrogen, Carlsbad, CA) and treated with DNase I (Invitrogen) to remove any possible genomic DNA contamination. Isolated RNA was stored at -80°C until utilization. Total DNA was isolated by back extraction using back extraction buffer (4 M guanidine thiocyanate, 50 mM sodium citrate, 1M Tris-base) from the inter- and organic phases of TRIzol extraction, followed by RNase A (Invitrogen) treatment to remove any possible contamination of RNA. Isolated DNA was stored at -20°C until further analysis.

### Mitochondrial DNA (mtDNA) sequencing

A 595-bp fragment of the mitochondrial NADH dehydrogenase 1 (*ND1*, 957 bp in length) was amplified and sequenced to determine genetic background of oocytes (“maternal”) used for cloning. The following PCR primers were designed based on the *Sus scrofa* mt DNA (GenBank access number AF034253): forward 5’-CCT ACT GGC CGT AGC ATT-3’, reverse 5’- GAA TCG TGG GTA TGA TGC TC-3’. PCR was performed using the *Taq* PCR kit (New England Biolab, Ipswich, MA). Briefly, 5 ng of total DNA in a 50 μl reaction mixture (1X standard *Taq* reaction buffer, 0.2 mM dNTP mixture, 0.75 μM of each primer, and 2.5 U *Taq* DNA Polymerase) was subjected to an initial denaturation at 94°C for 3 min, followed by 30 cycles of denaturation at 94°C for 20 sec, annealing at 60°C for 20 sec, extension at 72°C for 30 sec, and last extension at 72°C for 5 min. After purification by using ExoSAP-IT (USB, Cleveland, OH), the PCR products were sequenced by using the BigDye Terminator v1.1 Cycle Sequencing kit with ABI PRISM Model 3700 (Applied Biosystems Inc., Foster City, CA). Briefly, 30 μM of purified PCR products in a 20 μl reaction mixture (1X Terminator Ready Reaction Mix, 1X BigDye Sequencing Buffer, and 0.75 μM forward or reverse primer) were subjected to an initial denaturation at 94°C for 5 min, followed by 25 cycles of denaturation at 96°C for 10 sec, annealing at 50°C for 15 sec, and extension at 60°C for 4 min. The reaction mixture was then purified with DyeEx 2.0 (Qiagen, Valencia, CA), and electrophoresis was performed at Biotechnology/Bioservice Center at the University of Connecticut. Sequence similarity was measured by Basic Local Alignment Search Tool (BLAST, http://blast.ncbi.nlm.nih.gov/Blast.cgi). Bit score from the *in silico* sequence BLAST was used to determine maternal lineage by *ND1* sequence, and lineage-specific mtDNA polymorphisms were identified with Sequencher (version 4.1.4, Gene Codes, Ann Arbor, MI) by comparing with pig mtDNA reference sequences.

### DNA Microarray

The porcine Array-Ready Oligo Set (version 1.1, Operon Technologies, Alameda, CA) [24] was used in this study. Out of the 13,310 total probes, 566 genes were involved in mitochondrial functions including 534 and 32 genes from nuclear and mitochondrial DNA, respectively. Reference RNA was isolated and pooled from brain, kidney, liver, and lung tissues of male and female pig fetuses [25, 26]. Aminoallyl-linked cDNA was generated from 10 μg of the reference and extracted total RNA by reverse transcription, followed by labeling with Cy3 or Cy5, and hybridized to microarray slides as described [25, 27]. A total of 44 microarrays were used by co-hybridizing reference and extracted RNA with dye-swap. After scanning, local background subtraction was performed in GenePix Pro 6.0 (Molecular Devices, Union City, CA), and probes were flagged as “present” if they had at least 70% of feature pixels with more than two standard deviations (SD) above the background in either the Cy3 or Cy5 channel. Signal intensities of the “present probes” were loaded into GeneSpring 6.1 (Agilent Technologies, Palo Alto, CA). All microarrays were normalized by the Lowess method [28]. Probes were assigned as “informative” when they were present in either the reference or sample in more than 90% of the microarrays with raw expression values > 100, and a SD < 1.4 [29]. Informative probes were used for further analysis. Hierarchical clustering was conducted with three different informative probe sets: 1) all informative probes (global); informative probes involved in mitochondrial functions and encoded by the 2) nuclear DNA and 3) mtDNA. Differentially expressed (DE) genes were defined as those with a fold change ≥ 1.5 and a *p*-value ≤ 0.05 (Welch t-test adjusted by using Benjamini and Hochberg false discovery rate). Functional annotation was followed with RefSeq IDs from human orthologs of the DE genes using the Database for Annotation, Visualization and Integrated Discovery (DAVID, http://david.abcc.ncifcrf.gov) [30].

### Quantitative real-time RT-PCR (qPCR)

Nine genes involved in mitochondrial functions, of which 2 are encoded by mtDNA, were selected for qPCR validation. Using GenBank or *Sus Scrofa* Gene Index (SsGI) release 12.0, primers were designed to generate PCR products that harbored probe sequences of the microarray (Table 1). Tubulin beta-2 (*TBB2*) and the reference RNA were used as the internal control and as the calibrator, respectively. qPCR was performed with ABI 7500 Fast Real-Time PCR System (Applied Biosystems). Briefly, 2 ng of cDNA in a 20 μl reaction mixture [1X SYBR Green PCR master mix (Applied Biosystems), 0.3 μM of each primer] was subjected to an initial denaturation at 95°C for 10 min, followed by 40 cycles of denaturation at 95°C for 15 sec and annealing/extension at 60°C for 1 min. Data were analyzed using ABI prism SDS software (Applied Biosystems), and the relative expression levels of the target genes were calculated using the comparative C_T_ method according to the manufacturer’s instruction. Parametric data were tested for equal variance by using an F-test, and when it was not significant, data were subject to one-sided t-test (*p* < 0.05).

**Table 1:** Primer sequences for qPCR.

Gene	Type	Sequence	Tm	Amplicon (bp)	SsGI Release 12.0 accession number
*ACSL1*	Forward Primer	AGTCTTCACCCTGAATTATTCTCCAT	59	80	TC240600
	Reverse Primer	TTCCGAAGCTCTGGCCTTTT	60		
*CEBPA*	Forward Primer	GGACCACCAGTCTTGTCTGTACTG	59	80	TC292331
	Reverse Primer	TCCCTCTGGGATGGACTGATT	59		
*COX4I1*	Forward Primer	GCAGGATGTTGGCTACCAGAGT	59	92	TC275877
	Reverse Primer	CTTCACGACGCTTCCATGTG	59		
*COX7C*	Forward Primer	GCGCAATGTTGGGACAGAGT	60	79	TC298342
	Reverse Primer	TTCCCTGGACCCTCCTCATAG	59		
*GLUL*	Forward Primer	GGTGGGAAATGAGGTAGGAAAAT	58	81	TC162304
	Reverse Primer	CGAACATAATCAGGGCCTTTAGC	60		
*NDUFA4*	Forward Primer	GGAGGTACTGGAGCAGCACTGTAT	60	74	TC182398
	Reverse Primer	TTCTTCCTGTCCCAACAGACATC	59		
*NOTCH3*	Forward Primer	ACTTCACTGCATTCCAGATGGA	58	111	TC257056
	Reverse Primer	GCCAGTTCCCAAAGGGATTC	59		
*P38*	Forward Primer	GGGCCAAGGTGTCTCCATTT	60	96	TC287045
	Reverse Primer	CCTTCCTTCTCGCTCCAGTTG	60		
*TBB2*	Forward Primer	ACCCGAGGGACCTCTTTATTCA	60	84	TC279724
	Reverse Primer	AGACAGGAAGAAGAACAGATACACACA	59		
*UQCR*	Forward Primer	ATCTTCCTTCTTAAACTTGCCATTG	58	76	TC182529
	Reverse Primer	GCTGGTATGGGCCACTGACT	59		

## Results

### Determination of the “maternal background” of the cloned piglets

All microarray data from this study have been submitted to NCBI under the accession number of GSE68877. All sequences of mtDNA obtained in the current study were of either the Large White or the Duroc breed. For convenience, the *ND1* transcription start site was designated as Base #1 in the sequenced fragment. Seven breed-specific *ND1* gene polymorphisms between the Large White and Duroc (Table 2; T342C, T369C, T420C, G438A, G459A, C489A, and C712T from the Large White to the Duroc) were found in the 371-bp fragment analyzed. Alignment with mtDNA reference sequences confirmed lineage-specific *ND1* gene polymorphisms at the expected base positions as well. None of these are anticipated to cause translational variants.

**Table 2:** Mitochondrial *ND1* gene sequence analysis.

Group		Base comparison									BLAST hit	Matched strain	Bit score
Deceased	Base position	316	342	369	420	438	459	489	528	712			
	Reference	C	T^a^	T^a^	T^a^	G^a^	G^a^	C^a^	T	C^a^	AF486874	Large White	
	Control ID												
	263–1	C	T	T	T	G	G	C	C^b^	C	AF486874	Large White	1172
	263–2	C	T	T	T	G	G	C	C^b^	C	AF486874	Large White	1172
	263–3	C	T	T	T	G	G	C	C^b^	C	AF486874	Large White	1172
	263–4	C	T	T	T	G	G	C	C^b^	C	AF486874	Large White	1172
	263–5	C	T	T	T	G	G	C	C^b^	C	AF486874	Large White	1172
	Clone ID												
	628–1	C	T	T	T	G	G	C	C^b^	C	AF486874	Large White	1179
	629–1	C	T	T	T	G	G	C	C^b^	C	AF486874	Large White	1179
	635–1	C	T	T	T	G	G	C	C^b^	C	AF486874	Large White	1179
	635–2	C	C	C	C	A	A	A	T	T	AY337045	Duroc	1179
	637–1	C	T	T	T	G	G	C	C^b^	C	AF486874	Large White	1179
	642–1	C	C	C	C	A	A	A	T	T	AY337045	Duroc	1179
	671–5	C	C	C	C	A	A	A	T	T	AY337045	Duroc	1179
Live	Base position	316	342	369	420	438	459	489	528	712			
	Reference	C	C^a^	C^a^	C^a^	A^a^	A^a^	A^a^	T	T^a^	AY337045	Duroc	
	control ID												
	42–7	C	C	C	C	A	A	A	T	T	AY337045	Duroc	1179
	42–8	C	C	C	C	A	A	A	T	T	AY337045	Duroc	1179
	42–11	C	C	C	C	A	A	A	T	T	AY337045	Duroc	1179
	42–12	C	C	C	C	A	A	A	T	T	AY337045	Duroc	1179
	47–2	C	C	C	C	A	A	A	T	T	AY337045	Duroc	1179
	Clone ID												
	43–1	C	T	T	T	G	G	C	T	C	AF486874	Large White	1179
	43–2	C	T	T	T	G	G	C	T	C	AF486874	Large White	1179
	43–3	C	T	T	T	G	G	C	T	C	AF486874	Large White	1179
	44–1	T2	C	C	C	A	A	A	T	T	AY337045	Duroc	1172
	45–1	C	T	T	T	G	G	C	T	C	AF486874	Large White	1179

^a^Strain-specific DNA polymorphism

^b^Nonsense mutation

We then used the mtDNA sequences of the two breeds to determine the maternal background of the clones and their respective controls. The controls in the deceased and the live groups matched to the Large White (AF486874, bit score = 1,172), and Duroc (AY337045, bit score = 1,179), respectively. For the deceased clones, four gave the best match against the Large White (bit score = 1,179) and the other three against Duroc (bit score = 1,179). Four of the live clones matched to the Large White (bit score = 1,179) and the other to Duroc (bit score = 1,172).

### Hierarchical clustering of expression profiles of global, nuclear DNA- and mtDNA-encoded genes involved in mitochondrial functions

**Fig 1:**
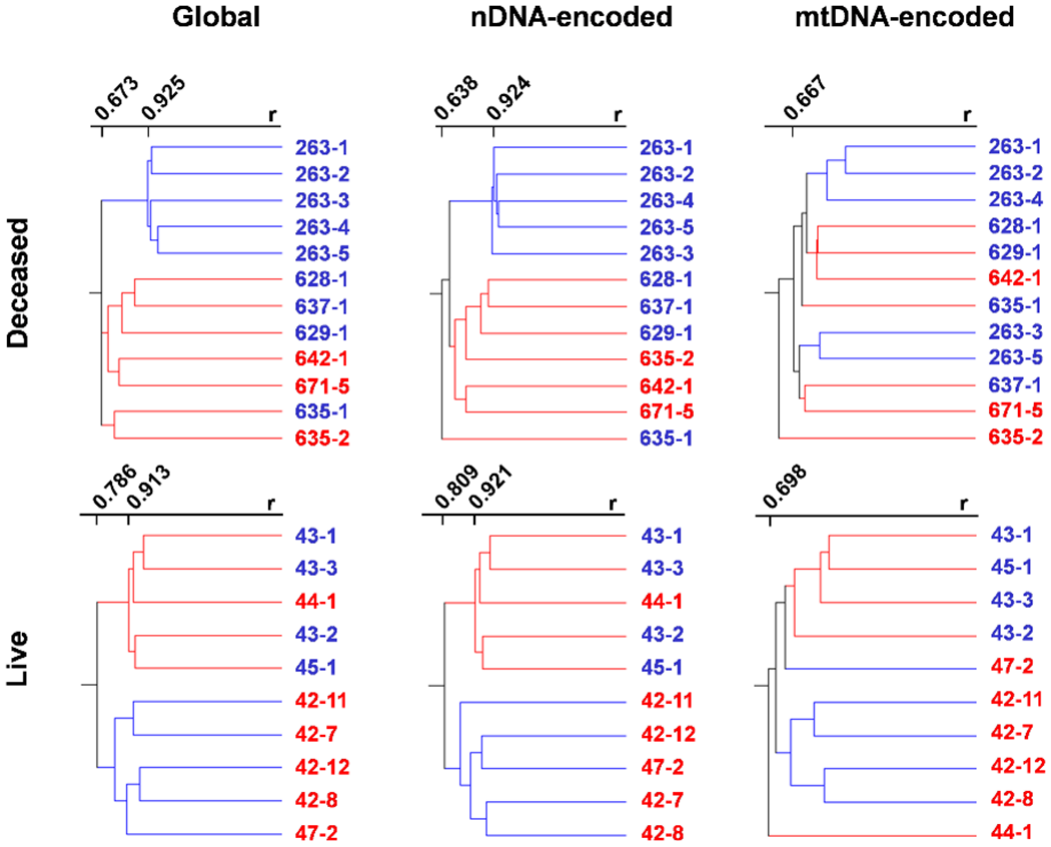
Hierarchical clustering of global, nuclear DNA (nDNA)- and mitochondrial DNA (mtDNA)-encoded genes of deceased (top panel) and live clones (bottom panel) vs. age-matched controls. From left to right, clustering with all informative probes, nuclear DNA- and mitochondrial DNA-encoded probes involved in mitochondrial functions were depicted. r = correlation coefficient, red line = clones, blue line = controls, red animal ID = Duroc, blue animal ID = Large White.

Expression profiles of global, nuclear DNA- and mtDNA-encoded genes of mitochondrial functions of the clones were compared with those of their conventionally bred counterparts (Fig 1). In the deceased group, global expression of 9,297 informative probes (69.8%) was largely clustered by treatment (clone vs. control). The correlation coefficient (r) of the controls was 0.925 compared with that of the clones at r = 0.673, demonstrating more variability in the cloned group. Likewise, 534 (4%) nuclear DNA-encoded genes of mitochondrial functions from the informative probes revealed that samples were clustered by treatment with r = 0.924 and 0.638 for controls and clones, respectively. Neither the global nor the nuclear DNA-encoded genes clustered with the maternal background. In contrast, the 32 (0.2%) mtDNA-encoded genes did not cluster by treatment or breed with the lowest correlation at r = 0.473.

In the live clone group, 9,955 probes (74.8%) were found to be informative for further analysis. As is with the dead group, hierarchical clustering of the informative probes also separated samples by treatment with r = 0.871 and 0.913 for controls and clones, respectively. Similarly, 451 nuclear DNA-encoded genes for mitochondrial functions (3.4%) were clustered by treatment with r = 0.879 and 0.921 for controls and clones, respectively. For the 32 mtDNA-encoded genes (0.2%), most samples separated by treatment with the exception of one clone of the Duroc background (Animal ID: 44–1) and a control of the Large White background (Animal ID: 47–2).

### Functional annotation of differentially expressed genes involved in mitochondrial functions

**Fig 2:**
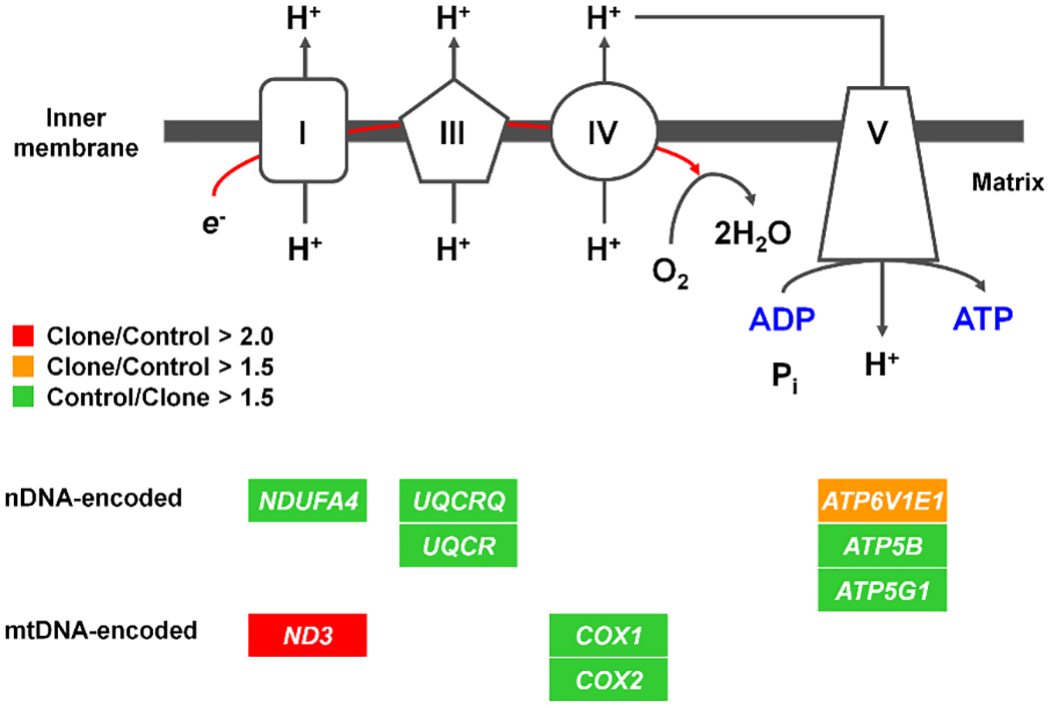
Dysregulated oxidative phosphorylation pathway of deceased cloned piglets. Genes in green boxes were down-regulated in clones (fold change > 1.5). Genes in orange box and red box were upregulated in clones by more than 1.5-, and 2.0-fold, respectively. Data were analyzed by using the Fisher’s exact test (*p* < 9.95 x 10^–7^, Benjamini and Hochberg FDR = 0.00019).

In the deceased group, 46 differentially expressed (DE) genes were identified, among which 43 were encoded by the nuclear DNA and 3 by the mtDNA. Seventeen of the nuclear-encoded genes were up-regulated and 26 down-regulated compared to controls (Table 3). Functional annotation of these DE genes revealed that the following nine involved in the OXPHOS pathway (Fig 2): NADH dehydrogenase (ubiquinone) 1 alpha subcomplex 4 (*NDUFA4*), ubiquinol-cytochrome c reductase complex III subunit VII (*UQCRQ*), ubiquinol-cytochrome c reductase (*UQCR*), ATPase V1 subunit E1 (*ATP6V1E1*), ATP synthase F1 complex beta polypeptide (*ATP5B*), and ATP synthase F0 complex subunit C1 (*ATP5G1*), all encoded by the nuclear DNA, as well as cytochrome c oxidase subunit 1 (*COX1*), cytochrome c oxidase subunit 2 (*COX2*), and NADH dehydrogenase 3 (*ND3*), all encoded by the mtDNA.

**Table 3:** Differentially expressed mitochondrial genes in deceased cloned piglets.

Encoded from	GenBank ID	TC ID	Fold change^c^	Gene Symbol	Gene Name
nDNA^a^	NM_004169	TC182203	3.65	SHMT1	SERINE HYDROXYMETHYLTRANSFERASE 1
	NM_004052	TC182058	2.64	BNIP3	BCL2/ADENOVIRUS E1B 19KDA INTERACTING PROTEIN 3
	NM_002065	TC162304	2.12	GLUL	GLUTAMATE-AMMONIA LIGASE (GLUTAMINE SYNTHETASE)
	NM_000817	TC189704	2.07	GAD1	GLUTAMATE DECARBOXYLASE 1
	NM_000274	TC181897	2.05	OAT	ORNITHINE AMINOTRANSFERASE
	NM_000663	TC186487	1.97	ABAT	4-AMINOBUTYRATE AMINOTRANSFERASE
	NM_002612	TC167221	1.91	PDK4	PYRUVATE DEHYDROGENASE KINASE, ISOZYME 4
	NM_001437	TC169575	1.88	ESR2	ESTROGEN RECEPTOR 2 (ER BETA)
	NM_001006642	TC166867	1.87	SLC25A25	SOLUTE CARRIER FAMILY 25 (MITOCHONDRIAL CARRIER; PHOSPHATE CARRIER), MEMBER 25
	NM_021232	N/A	1.73	PRODH2	PROLINE DEHYDROGENASE (OXIDASE) 2
	NM_001696	TC162504	1.62	ATP6V1E1	ATPASE, H+ TRANSPORTING, LYSOSOMAL 31KDA, V1 SUBUNIT E1
	NM_000349	TC162876	1.61	STAR	STEROIDOGENIC ACUTE REGULATOR
	NM_012094	TC164220	1.57	PRDX5	PEROXIREDOXIN 5
	NM_022036	TC164081	1.56	GPRC5C	G PROTEIN-COUPLED RECEPTOR, FAMILY C, GROUP 5, MEMBER C
	NM_020166	TC186613	1.55	MCCC1	METHYLCROTONOYL-COENZYME A CARBOXYLASE 1 (ALPHA)
	NM_004415	TC188422	1.53	DSP	DESMOPLAKIN
	NM_005763	TC190934	1.52	AASS	AMINOADIPATE-SEMIALDEHYDE SYNTHASE
	NM_002080	TC163473	0.67	GOT2	GLUTAMIC-OXALOACETIC TRANSAMINASE 2, MITOCHONDRIAL (ASPARTATE AMINOTRANSFERASE 2)
	NM_006111	TC185178	0.66	ACAA2	ACETYL-COENZYME A ACYLTRANSFERASE 2 (MITOCHONDRIAL 3-OXOACYL-COENZYME A THIOLASE)
	NM_014297	TC181027	0.66	ETHE1	ETHYLMALONIC ENCEPHALOPATHY 1
	NM_000308	TC182001	0.66	PPGB	PROTECTIVE PROTEIN FOR BETA-GALACTOSIDASE
	NM_000397	TC163629	0.66	CYBB	CYTOCHROME B-245, BETA POLYPEPTIDE
	NM_006793	TC181710	0.65	PRDX3	PEROXIREDOXIN 3
	NM_003679	TC168142	0.65	KMO	KYNURENINE 3-MONOOXYGENASE (KYNURENINE 3-HYDROXYLASE)
	NM_015469	TC188504	0.65	NIPSNAP3A	NIPSNAP HOMOLOG 3A
	NM_000016	TC183520	0.64	ACADM	ACYL-COENZYME A DEHYDROGENASE, C-4 TO C-12 STRAIGHT CHAIN
	NM_001686	TC162934	0.64	ATP5B	ATP SYNTHASE, H+ TRANSPORTING, MITOCHONDRIAL F1 COMPLEX, BETA POLYPEPTIDE
	NM_020312	TC182787	0.64	COQ9	COENZYME Q9 HOMOLOG
	NM_012259	TC168760	0.64	HEY2	HAIRY/ENHANCER-OF-SPLIT RELATED WITH YRPW MOTIF 2
	NM_002489	TC182398	0.64	NDUFA4	NADH DEHYDROGENASE (UBIQUINONE) 1 ALPHA SUBCOMPLEX, 4, 9KDA
	NM_014402	TC164365	0.63	UQCRQ	UBIQUINOL-CYTOCHROME C REDUCTASE, COMPLEX III SUBUNIT VII, 9.5KDA
	NM_002396	TC167924	0.63	ME2	MALIC ENZYME 2, NAD(+)-DEPENDENT, MITOCHONDRIAL
	NM_001985	TC163232	0.62	ETFB	ELECTRON-TRANSFER-FLAVOPROTEIN, BETA POLYPEPTIDE
	NM_014481	TC166660	0.62	APEX2	APEX NUCLEASE (APURINIC/APYRIMIDINIC ENDONUCLEASE) 2
	NM_006830	TC182529	0.62	UQCR	UBIQUINOL-CYTOCHROME C REDUCTASE, 6.4KDA SUBUNIT
	NM_020548	TC182240	0.61	DBI	DIAZEPAM BINDING INHIBITOR (GABA RECEPTOR MODULATOR, ACYL-COENZYME A BINDING PROTEIN)
	NM_012343	TC189901	0.58	NNT	NICOTINAMIDE NUCLEOTIDE TRANSHYDROGENASE
	NM_016497	TC165872	0.55	MRPL51	MITOCHONDRIAL RIBOSOMAL PROTEIN L51
	NM_005175	TC163315	0.55	ATP5G1	ATP SYNTHASE, H+ TRANSPORTING, MITOCHONDRIAL F0 COMPLEX, SUBUNIT C1 (SUBUNIT 9)
	NM_002979	TC163162	0.54	SCP2	STEROL CARRIER PROTEIN 2
	NM_006082	TC181275	0.52	TUBA6	TUBULIN, ALPHA, UBIQUITOUS
	NM_001876	TC164450	0.46	CPT1A	CARNITINE PALMITOYLTRANSFERASE 1A
	NM_001948	TC184267	0.45	DUT	DUTP PYROPHOSPHATASE
mtDNA^b^	NM_006818	TC168152	0.65	COX1	CYTOCHOROME C OXIDASE SUBUNIT 1
	N/A	TC181301	0.65	COX2	CYTOCHROME C OXIDASE SUBUNIT 2
	N/A	TC190307	2.11	ND3	NADH DEHYDROGENASE 3

^a^nDNA: nuclear DNA

^b^mtDNA: mitochondrial DNA

^c^Fold change = clone/age-matched control

In the live clones, 35 nuclear DNA-encoded DE genes were found (Table 4). Among them, 24 were up-regulated and 11 were down-regulated compared to controls. Three mtDNA-encoded genes were also identified as differentially expressed. Functional analysis revealed that six nuclear genes were involved in the “FATTY ACID METABOLISM” pathway. These are dodecenoyl-coenzyme A delta isomerase (*DCI*), enoyl coenzyme A hydratase short chain 1 (*ECHS1*), glutaryl-coenzyme A dehydrogenase (*GCDH*), L-3-hydrozyacyl-coenzyme A dehydrogenase short chain (*HADHSC*), acetyl-coenzyme A acyltransferase 2 (*ACAA2*), and fatty-acid-coenzyme A ligase long chain 1 (*ACSL1*). However, no significant functional annotation was enriched with the DE genes encoded by mtDNA.

**Table 4:** Differentially expressed mitochondrial genes in live cloned piglets.

Encoded from	GenBank ID	TC ID	Fold change^c^	Gene Symbol	Gene Name
nDNA^a^	NM_014585	TC165023	2.13	SLC40A1	SOLUTE CARRIER FAMILY 40 (IRON-REGULATED TRANSPORTER), MEMBER 1
	NM_004331	TC184073	1.79	BNIP3L	BCL2/ADENOVIRUS E1B 19KDA INTERACTING PROTEIN 3-LIKE
	NM_170711	TC183283	1.70	DAZAP1	DAZ ASSOCIATED PROTEIN 1
	NM_001752	TC181995	1.69	CAT	CATALASE
	NM_012343	TC189901	1.65	NNT	NICOTINAMIDE NUCLEOTIDE TRANSHYDROGENASE
	NM_001919	TC181889	1.64	DCI	DODECENOYL-COENZYME A DELTA ISOMERASE (3,2 TRANS-ENOYL-COENZYME A ISOMERASE)
	NM_015917	TC161988	1.63	GSTK1	GLUTATHIONE S-TRANSFERASE SUBUNIT 13 HOMOLOG
	NM_002979	TC163162	1.63	SCP2	STEROL CARRIER PROTEIN 2
	NM_000456	TC185923	1.61	SUOX	SULFITE OXIDASE
	NM_005881	TC163821	1.59	BCKDK	BRANCHED CHAIN KETOACID DEHYDROGENASE KINASE
	NM_018838	TC164907	1.58	NDUFA12	NADH DEHYDROGENASE (UBIQUINONE) 1 ALPHA SUBCOMPLEX, 12
	NM_004092	TC181253	1.57	ECHS1	ENOYL COENZYME A HYDRATASE, SHORT CHAIN, 1, MITOCHONDRIAL
	NM_000159	TC182620	1.57	GCDH	GLUTARYL-COENZYME A DEHYDROGENASE
	NM_005327	TC183224	1.56	HADHSC	L-3-HYDROXYACYL-COENZYME A DEHYDROGENASE, SHORT CHAIN
	NM_005984	TC163540	1.56	SLC25A1	SOLUTE CARRIER FAMILY 25 (MITOCHONDRIAL CARRIER; CITRATE TRANSPORTER), MEMBER 1
	NM_000398	TC183476	1.55	CYB5R3	CYTOCHROME B5 REDUCTASE 3
	NM_006111	TC185178	1.54	ACAA2	ACETYL-COENZYME A ACYLTRANSFERASE 2 (MITOCHONDRIAL 3-OXOACYL-COENZYME A THIOLASE)
	NM_004453	TC166131	1.53	ETFDH	ELECTRON-TRANSFERRING-FLAVOPROTEIN DEHYDROGENASE
	NM_001359	TC182911	1.53	DECR1	2,4-DIENOYL COA REDUCTASE 1, MITOCHONDRIAL
	NM_001609	TC167600	1.52	ACADSB	ACYL-COENZYME A DEHYDROGENASE, SHORT/BRANCHED CHAIN
	NM_032549	TC167347	1.51	IMMP2L	IMP2 INNER MITOCHONDRIAL MEMBRANE PEPTIDASE-LIKE
	NM_002858	TC166623	1.51	ABCD3	ATP-BINDING CASSETTE, SUB-FAMILY D (ALD), MEMBER 3
	NM_012181	TC161966	1.50	FKBP8	FK506 BINDING PROTEIN 8, 38KDA
	NM_030791	TC164539	1.50	SGPP1	SPHINGOSINE-1-PHOSPHATE PHOSPHATASE 1
	NM_018947	TC163123	0.65	CYCS	CYTOCHROME C, SOMATIC
	NM_001995	TC163394	0.64	ACSL1	FATTY-ACID-COENZYME A LIGASE, LONG-CHAIN 1
	NM_018188	TC162178	0.63	ATAD3A	ATPASE FAMILY, AAA DOMAIN CONTAINING 3A
	NM_005729	TC182963	0.60	PPIF	PEPTIDYLPROLYL ISOMERASE F (CYCLOPHILIN F)
	NM_031212	TC182541	0.58	SLC25A28	SOLUTE CARRIER FAMILY 25, MEMBER 28
	NM_000032	TC166637	0.58	ALAS2	AMINOLEVULINATE, DELTA-, SYNTHASE 2
	NM_002065	TC165015	0.54	GLUL	GLUTAMATE-AMMONIA LIGASE (GLUTAMINE SYNTHETASE)
	NM_001006642	TC166867	0.48	SLC25A25	SOLUTE CARRIER FAMILY 25 (MITOCHONDRIAL CARRIER; PHOSPHATE CARRIER), MEMBER 25
	NM_199187	TC162826	0.41	KRT18	KERATIN 18
	NM_000636	TC164641	0.25	SOD2	SUPEROXIDE DISMUTASE 2, MITOCHONDRIAL
	NM_002612	N/A	0.20	PDK4	PYRUVATE DEHYDROGENASE KINASE, ISOZYME 4
mtDNA^b^	N/A	TC176636	1.60	ATP6	ATPASE SUBUNIT 6
	N/A	TC166635	0.66	COX3	CYTOCHROME C OXIDASE SUBUNIT 3
	NM_016357	TC167974	0.62	ND2	NADH DEHYDROGENASE SUBUNIT 2

^a^nDNA: nuclear DNA

^b^mtDNA: mitochondrial DNA

^c^Fold change = clone/age-matched control

**Fig 3:**
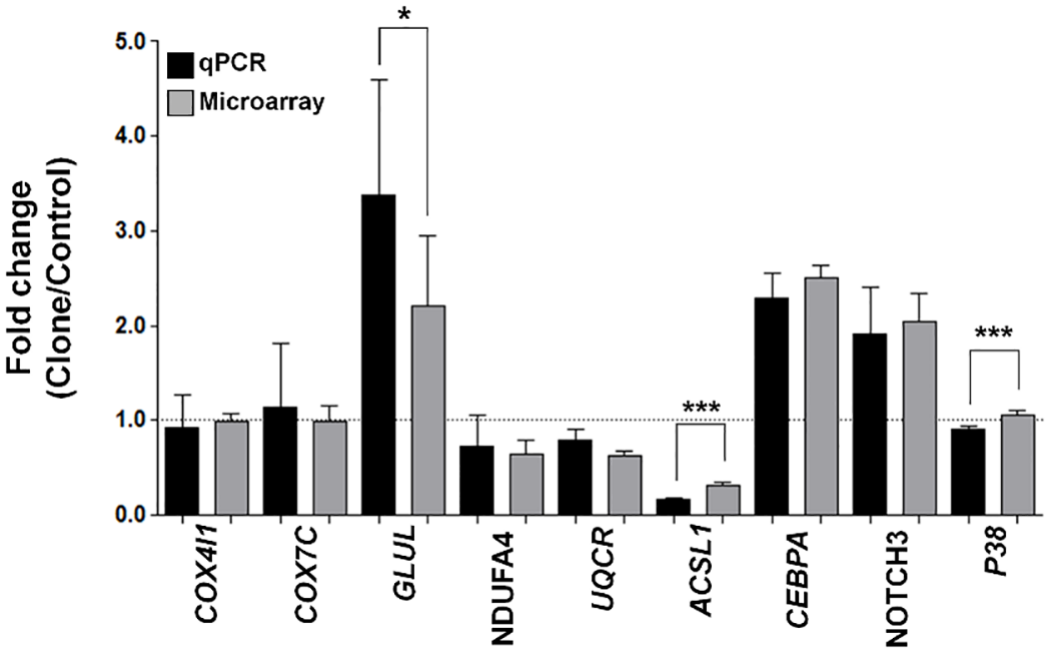
Comparisons of representative gene expressions from qPCR and microarray. Black bars indicate mean gene expression data from qPCR, and gray bars from microarray. Error bars mean ± SD. Data were analyzed by one-sided t-test (*p < 0.05, ***p < 0.001).

The expression levels of nine genes were validated by qPCR (Fig 3). They are *GLUL*, *CEBPA*, *NOTCH3* (up-regulated in clones), *NDUFA4*, *UQCR*, *ACSL1* (down-regulated in clones), and *COX4l1*, *COX7C*, *P38* (control level of expression in clones). Although slight differences in the fold changes of three genes were observed (*GLUL*, *ACSL1*, *and P38*), overall the qPCR results confirmed the directionality of the microarray.

## Discussion

Using sequence variations in the mtDNA-encoded *ND1* gene, we first determined the “maternal background” of clones and controls used in this study and subsequently analyzed if expression of genes involved in mitochondrial functions were normally expressed in the deceased and the live clones or if they are related to their maternal lineages. We found that all cloned piglets had either the Large White or the Duroc as the maternal background. These breeds are the most common commercial pig lineages of the world and serve to produce many mixed-lineaged commercial hogs [31]. Although these European domestic pig lineages are closely related in their maternal lines, Large White is supposed to have separated from the European lineages around 58,000 year ago. In addition, the genetic distance between the Large White and the Duroc is 40-fold longer than that among European domestic lineages [32]; therefore, it should be feasible to distinguish between the maternal lineage of the Large White and the Duroc by using mitochondrial *ND 1* gene sequence By sequence alignment, we found seven lineage-specific *ND1* polymorphisms from the 371-bp target fragment. Interestingly, these polymorphisms were not supposed to induce translational variants. Therefore, these *ND1* polymorphisms are good candidates for lineage determination while not affecting function s of the mitochondrial OXPHOS complex I. We did not find maternal lineage-dependent survivability of cloned piglets. Whether oocytes of different maternal breed background critically impact the cloning efficiency or survivability in pigs requires additional study and larger datasets.

The persistence of donor cell-derived mtDNA in cloned animals has been reported in four mammalian species including pigs [9]. The degree of heteroplasmy; however, has been found to be highly variable, ranging from 0 to 57% when donor cell was an embryonic blastomere determined by restriction digestion and Southern blotting [10]. Most somatic cell clones only contain a low fraction (<1%) of the donor cell mtDNA as determined by the more sensitive qPCR method [11]. In general, it is thought that the onset of physiological effects of pathogenic heteroplasmy begins when the ratio of mutant to normal mtDNA exceeds a specific threshold. For example, heteroplasmy-induced mitochondrial dysfunction contributes to pulmonary hypertension associated with cardiac output in mice. Quantification analysis revealed that most organs in these mice have more than 30% of mutant mtDNA [12]. In addition, a very low proportion of mutant heteroplasmy (less than 5%) is of questionable clinical significance since it may be due to laboratory error [33]. In preliminary trials of this study, we determined that a higher than 25% heteroplasmy would be detectable by sequencing the *ND1*. None of the cloned animals has such a high level of heteroplasmy (data not shown). Therefore, we excluded the possibility of pathogenesis contributed by donor cell-derived mtDNA.

Developmental competence of reconstructed embryos is significantly affected by the cytoplasm of the recipient oocytes. For example, oocytes from the hybrid strain BDF1 produce the highest cloning efficiency in the mouse [34]. Using oocytes of three different breeds and the same donor cell line, both transferable cloned embryos and viable bovine cloned fetuses were found to vary in phenotype based on different oocyte cytoplasm [19]. These cytoplasmic effects continued to affect birth weight, crump-rump length, and femoral length of cloned fetuses. In inter-subspecies SCNT embryos in goats, full term birth rates were highest in intra-species SCNT controls, followed by NT using recipient oocytes of crossbred goats with the lowest birth rate resulting from Boer donor cells into Saanen oocytes [20]. Furthermore, structure and quantity of mitochondria in the recipient oocytes have also been shown to influence the developmental potential of reconstructed bovine embryos [35]. For instance, oocytes with different mtDNA haplotype have a different copy number of mtDNA and ATP content. These oocytes have differential expression levels of *COX1* and *COX3*, key components of the OXPHOS pathway and ATP generation, which is suspected to affect the cloning efficiency. These results shed light on the relevance of the maternal oocyte lineage to generate cloned animals by SCNT. In contrast, the maternal lineages revealed in this study were found to not be critical for embryo-fetal development or at least for viability of cloned piglets. Therefore, it is likely that the recipient oocytes retrieved at least from the Large White and/or the Duroc do not affect the cloning efficiency in pigs as opposed to observations in the mouse or bovine where the source of the oocyte is important.

Despite the lack of correlation of clone pig survival and mitochondrial lineages, gene expression analyses revealed an interruption of the interaction between genes encoded by the nuclear and mtDNA. Lack of expression coordination, especially abnormal expression of mtDNA-encoded genes, can affect nuclear DNA expression at the cellular level. For example, in the respiratory-deficient cell line lacking mtDNA (ρ°cells), the expression levels of numerous nuclear DNA-encoded genes for mitochondrial biogenesis and functions are dysregulated [36]. Kulawiec et al. reported that in ρ°cells, *UQCRC1*, a nuclear DNA-encoded gene, is down-regulated by as much as 10-fold. Furthermore, in both breast and ovarian carcinomas, *UQCRC1* expression is positively correlated with mtDNA-encoded *COX2* expression [17]. In this study, expression profiles of mtDNA-encoded genes in the live clone group showed treatment-dependent clustering. However, clustering analysis based on the expression profiles of mtDNA-encoded genes of the deceased clones showed an intermixed pattern with their age-matched control. These results imply that disruption in mitochondria-to-nucleus interaction occurred only in the deceased cloned piglets. Such lack of coordination may affect energy metabolism in the lungs and contribute to the overall deficiency in full development of clones.

Annotation analysis revealed that defects in the OXPHOS pathway could have contributed early death of cloned piglets. Several genes for OXPHOS pathway were differentially expressed in the deceased cloned piglets. For example, *COX1* and *COX2*, two of the three mtDNA-encoded genes involved in OXPHOS complex IV, were significantly down-regulated only in the deceased clones. NDUFA4 and ND3 are complex I subunits encoded from nuclear and mtDNA, respectively. Mitochondrial genes like *ND3* are down-regulated under pathological conditions such as glioblastoma [37] and hypoxia [38], and it was accompanied by the reduction of the activities of the respiratory enzyme complexes. Furthermore, down-regulation of these genes leads to loss of complex IV and complex I assemblies and activities [39, 40]. Another down-regulated gene in clones, *UQCRQ*, encodes a ubiquinone-binding protein (QP-C) as a subunit of complex III. A deletion in this gene decreases cytochrome b content which affects the assembly or maintenance of complex III [41]. ATP5B is a beta subunit of ATP synthase that catalyzes ATP formation using the energy of proton flux through the mitochondrial inner membrane during OXPHOS [42], and it is down-regulated in ulcerative colitis affecting the colon mucosa with other mitochondrial proteins [43]. Multiple copies of subunit C in the transmembrane portion (F0) of the ATP synthase transport rotate protons across the mitochondrial inner membrane to the F1-ATPase. *ATP5G1* is one of the subunit C genes, and mature forms of ATP5G1, ATP5G2, and ATP5G3 are identical [44]. In alcohol consuming rats, all of these genes were dysregulated accompanying mitochondrial injury in the pancreas [45]. The abnormal expression of all of the genes above in deceased clones likely resulted in reduced ATP generation and pulmonary functionality.

In conclusion, this study demonstrates that the lineage of recipient oocytes may have little or no effect on the viability of cloned piglets by SCNT. However, cloned pigs may develop abnormal expression of both nuclear and mtDNA-encoded genes especially in the OXPHOS pathway, leading to a disruption of coordination of nuclear and mitochondrion interactions.

## References

[1] Wilmut I, Schnieke AE, McWhir J, Kind AJ, Campbell KH. Viable offspring derived from fetal and adult mammalian cells. Nature. 1997;385(6619):810–3. . 9039911 10.1038/385810a0

[2] Kishigami S, Wakayama S, Hosoi Y, Iritani A, Wakayama T. Somatic cell nuclear transfer: infinite reproduction of a unique diploid genome. Exp Cell Res. 2008;314(9):1945–50. 10.1016/j.yexcr.2008.01.027 18346729

[3] Ogonuki N, Inoue K, Yamamoto Y, Noguchi Y, Tanemura K, Suzuki O, et al Early death of mice cloned from somatic cells. Nat Genet. 2002;30(3):253–4. . 11836501 10.1038/ng841

[4] Lai L, Kolber-Simonds D, Park KW, Cheong HT, Greenstein JL, Im GS, et al Production of alpha-1,3-galactosyltransferase knockout pigs by nuclear transfer cloning. Science. 2002;295(5557):1089–92. . 11778012 10.1126/science.1068228

[5] Lan GC, Chang ZL, Luo MJ, Jiang YL, Han D, Wu YG, et al Production of cloned goats by nuclear transfer of cumulus cells and long-term cultured fetal fibroblast cells into abattoir-derived oocytes. Molecular reproduction and development. 2006;73(7):834–40. . 16572465 10.1002/mrd.20443

[6] Powell AM, Talbot NC, Wells KD, Kerr DE, Pursel VG, Wall RJ. Cell donor influences success of producing cattle by somatic cell nuclear transfer. Biology of reproduction. 2004;71(1):210–6. . 14998911 10.1095/biolreprod.104.027193

[7] Urakawa M, Ideta A, Sawada T, Aoyagi Y. Examination of a modified cell cycle synchronization method and bovine nuclear transfer using synchronized early G1 phase fibroblast cells. Theriogenology. 2004;62(3–4):714–28. . 15226025 10.1016/j.theriogenology.2003.11.024

[8] Wallace DC. Mitochondrial diseases in man and mouse. Science. 1999;283(5407):1482–8. . 10066162 10.1126/science.283.5407.1482

[9] Burgstaller JP, Schinogl P, Dinnyes A, Muller M, Steinborn R. Mitochondrial DNA heteroplasmy in ovine fetuses and sheep cloned by somatic cell nuclear transfer. BMC developmental biology. 2007;7:141 . 18154666 10.1186/1471-213X-7-141 PMC2323970

[10] Hiendleder S, Schmutz SM, Erhardt G, Green RD, Plante Y. Transmitochondrial differences and varying levels of heteroplasmy in nuclear transfer cloned cattle. Molecular reproduction and development. 1999;54(1):24–31. 10.1002/(SICI)1098-2795(199909)54:1<24::AID-MRD4>3.0.CO;2-S . 10423294

[11] Hiendleder S, Zakhartchenko V, Wenigerkind H, Reichenbach HD, Bruggerhoff K, Prelle K, et al Heteroplasmy in bovine fetuses produced by intra- and inter-subspecific somatic cell nuclear transfer: neutral segregation of nuclear donor mitochondrial DNA in various tissues and evidence for recipient cow mitochondria in fetal blood. Biology of reproduction. 2003;68(1):159–66. . 12493708 10.1095/biolreprod.102.008201

[12] Acton BM, Lai I, Shang X, Jurisicova A, Casper RF. Neutral mitochondrial heteroplasmy alters physiological function in mice. Biology of reproduction. 2007;77(3):569–76. . 17554081 10.1095/biolreprod.107.060806

[13] Neupert W. Protein import into mitochondria. Annu Rev Biochem. 1997;66:863–917. . 9242927 10.1146/annurev.biochem.66.1.863

[14] Cotter D, Guda P, Fahy E, Subramaniam S. MitoProteome: mitochondrial protein sequence database and annotation system. Nucleic acids research. 2004;32(Database issue):D463–7. 10.1093/nar/gkh048 14681458 PMC308782

[15] Butow RA, Avadhani NG. Mitochondrial signaling: the retrograde response. Mol Cell. 2004;14(1):1–15. . 15068799 10.1016/s1097-2765(04)00179-0

[16] McKenzie M, Trounce I. Expression of Rattus norvegicus mtDNA in Mus musculus cells results in multiple respiratory chain defects. J Biol Chem. 2000;275(40):31514–9. . 10908563 10.1074/jbc.M004070200

[17] Kulawiec M, Arnouk H, Desouki MM, Kazim L, Still I, Singh KK. Proteomic analysis of mitochondria-to-nucleus retrograde response in human cancer. Cancer Biol Ther. 2006;5(8):967–75. . 16775426 10.4161/cbt.5.8.2880

[18] Bruggerhoff K, Zakhartchenko V, Wenigerkind H, Reichenbach HD, Prelle K, Schernthaner W, et al Bovine somatic cell nuclear transfer using recipient oocytes recovered by ovum pick-up: effect of maternal lineage of oocyte donors. Biology of reproduction. 2002;66(2):367–73. . 11804950 10.1095/biolreprod66.2.367

[19] Hiendleder S, Prelle K, Bruggerhoff K, Reichenbach HD, Wenigerkind H, Bebbere D, et al Nuclear-cytoplasmic interactions affect in utero developmental capacity, phenotype, and cellular metabolism of bovine nuclear transfer fetuses. Biology of reproduction. 2004;70(4):1196–205. . 14681199 10.1095/biolreprod.103.023028

[20] Jian-Quan C, Juan C, Xu-Jun X, Guo-Hui L, Si-Guo L, Hong-Ying S, et al Effect of cytoplast on the development of inter-subspecies nuclear transfer reconstructed goat embryo. Molecular reproduction and development. 2007;74(5):568–73. . 17039506 10.1002/mrd.20647

[21] Yan ZH, Zhou YY, Fu J, Jiao F, Zhao LW, Guan PF, et al Donor-host mitochondrial compatibility improves efficiency of bovine somatic cell nuclear transfer. BMC developmental biology. 2010;10:31 10.1186/1471-213X-10-31 20302653 PMC2858029

[22] Lee JH, Peters A, Fisher P, Bowles EJ, St John JC, Campbell KH. Generation of mtDNA homoplasmic cloned lambs. Cellular reprogramming. 2010;12(3):347–55. 10.1089/cell.2009.0096 . 20698774

[23] Carroll JA, Carter DB, Korte SW, Prather RS. Evaluation of the acute phase response in cloned pigs following a lipopolysaccharide challenge. Domestic animal endocrinology. 2005;29(3):564–72. 10.1016/j.domaniend.2005.03.009 . 16153505

[24] Zhao SH, Recknor J, Lunney JK, Nettleton D, Kuhar D, Orley S, et al Validation of a first-generation long-oligonucleotide microarray for transcriptional profiling in the pig. Genomics. 2005;86(5):618–25. . 16216716 10.1016/j.ygeno.2005.08.001

[25] Park J, Marjani SL, Lai L, Samuel M, Wax D, Davis SR, et al Altered gene expression profiles in the brain, kidney, and lung of deceased neonatal cloned pigs. Cellular reprogramming. 2010;12(5):589–97. 10.1089/cell.2010.0004 20726773 PMC2998943

[26] Park J, Marjani SL, Lai L, Samuel M, Wax D, Davis SR, et al Altered gene expression profiles in the brain, kidney, and lung of deceased neonatal cloned pigs. unpublished. 10.1089/cell.2010.0004 20726773 PMC2998943

[27] Park J, Lai L, Samuel M, Wax D, Bruno RS, French R, et al Altered gene expression profiles in the brain, kidney, and lung of one-month-old cloned pigs. Cellular reprogramming. 2011;13(3):215–23. 10.1089/cell.2010.0088 21453050 PMC3104288

[28] Clevel WS, and Devlin S. J.. Locally-weighted regression: an approach to regression analysis by local fitting. J Am Stat Assoc. 1988;83:596–610.

[29] Jensen KB, Watt FM. Single-cell expression profiling of human epidermal stem and transit-amplifying cells: Lrig1 is a regulator of stem cell quiescence. Proc Natl Acad Sci U S A. 2006;103(32):11958–63. . 16877544 10.1073/pnas.0601886103 PMC1567680

[30] Dennis G Jr., Sherman BT, Hosack DA, Yang J, Gao W, Lane HC, et al DAVID: Database for Annotation, Visualization, and Integrated Discovery. Genome Biol. 2003;4(5):P3 . 12734009 PMC193660

[31] Tagliaro CH, Franco MHLP, Schneider MPC, de Brito BG, Barbosa AS. Biochemical polymorphisms and genetic relationships between brazilian and foreign breeds of pigs reared in Brazil. Ciencia Rural, Santa Maria. 1999;29(2):319.

[32] Kim KI, Lee JH, Li K, Zhang YP, Lee SS, Gongora J, et al Phylogenetic relationships of Asian and European pig breeds determined by mitochondrial DNA D-loop sequence polymorphism. Anim Genet. 2002;33(1):19–25. . 11849133 10.1046/j.1365-2052.2002.00784.x

[33] Wong LJ, Boles RG. Mitochondrial DNA analysis in clinical laboratory diagnostics. Clin Chim Acta. 2005;354(1–2):1–20. . 15748595 10.1016/j.cccn.2004.11.003

[34] Latham KE. Strain-specific differences in mouse oocytes and their contributions to epigenetic inheritance. Development. 1994;120(12):3419–26. . 7821212 10.1242/dev.120.12.3419

[35] Jiao F, Yan JB, Yang XY, Li H, Wang Q, Huang SZ, et al Effect of oocyte mitochondrial DNA haplotype on bovine somatic cell nuclear transfer efficiency. Molecular reproduction and development. 2007;74(10):1278–86. . 17290429 10.1002/mrd.20698

[36] Traven A, Wong JM, Xu D, Sopta M, Ingles CJ. Interorganellar communication. Altered nuclear gene expression profiles in a yeast mitochondrial dna mutant. J Biol Chem. 2001;276(6):4020–7. . 11054416 10.1074/jbc.M006807200

[37] Dmitrenko V, Shostak K, Boyko O, Khomenko O, Rozumenko V, Malisheva T, et al Reduction of the transcription level of the mitochondrial genome in human glioblastoma. Cancer Lett. 2005;218(1):99–107. . 15639345 10.1016/j.canlet.2004.07.001

[38] Piruat JI, Lopez-Barneo J. Oxygen tension regulates mitochondrial DNA-encoded complex I gene expression. J Biol Chem. 2005;280(52):42676–84. . 16257962 10.1074/jbc.M507044200

[39] Li Y, D'Aurelio M, Deng JH, Park JS, Manfredi G, Hu P, et al An assembled complex IV maintains the stability and activity of complex I in mammalian mitochondria. J Biol Chem. 2007 . 17452320 10.1074/jbc.M701056200

[40] Dietrich JB, Poirier R, Aunis D, Zwiller J. Cocaine downregulates the expression of the mitochondrial genome in rat brain. Ann N Y Acad Sci. 2004;1025:345–50. . 15542735 10.1196/annals.1316.042

[41] Haut S, Brivet M, Touati G, Rustin P, Lebon S, Garcia-Cazorla A, et al A deletion in the human QP-C gene causes a complex III deficiency resulting in hypoglycaemia and lactic acidosis. Hum Genet. 2003;113(2):118–22. . 12709789 10.1007/s00439-003-0946-0

[42] Ohta S, Tomura H, Matsuda K, Kagawa Y. Gene structure of the human mitochondrial adenosine triphosphate synthase beta subunit. J Biol Chem. 1988;263(23):11257–62. . 2900241

[43] Hsieh SY, Shih TC, Yeh CY, Lin CJ, Chou YY, Lee YS. Comparative proteomic studies on the pathogenesis of human ulcerative colitis. Proteomics. 2006;6(19):5322–31. . 16947118 10.1002/pmic.200500541

[44] Yan WL, Lerner TJ, Haines JL, Gusella JF. Sequence analysis and mapping of a novel human mitochondrial ATP synthase subunit 9 cDNA (ATP5G3). Genomics. 1994;24(2):375–7. . 7698763 10.1006/geno.1994.1631

[45] Li HS, Zhang JY, Thompson BS, Deng XY, Ford ME, Wood PG, et al Rat mitochondrial ATP synthase ATP5G3: cloning and upregulation in pancreas after chronic ethanol feeding. Physiol Genomics. 2001;6(2):91–8. . 11459924 10.1152/physiolgenomics.2001.6.2.91

